# Silencing of insulin receptor substrate–1 increases cell death in retinal Müller cells

**Published:** 2012-02-01

**Authors:** Robert J. Walker, Nancy M. Anderson, Suleiman Bahouth, Jena J. Steinle

**Affiliations:** 1 Department of Ophthalmology, Hamilton Eye Institute, University of Tennessee Health Science Center, Memphis, TN; 2 Department of Anatomy & Neurobiology, University of Tennessee Health Science Center, Memphis, TN; 3 Department of Pharmacology, University of Tennessee Health Science Center, Memphis, TN

## Abstract

**Purpose:**

To determine whether β-adrenergic receptors require insulin receptor substrate (IRS)-1 activity to regulate apoptosis in retinal Müller cells.

**Methods:**

Müller cells were cultured in Dulbecco's Modified Eagle Medium (DMEM) medium grown in normal (5 mm) or high glucose (25 mM) conditions. The medium was supplemented with 10% fetal bovine serum and antibiotics. Cells were allowed to reach 80%–90% confluence. After becoming appropriately confluent, cells were placed in medium with reduced serum (2%) for 18–24 h to eliminate any effects of fetal bovine serum. Cells were then transfected with 10 ug of *IRS-1* small hairpin RNA (shRNA). Forty-eight hours following transfection, cells were lysed and harvested for protein analysis using western blotting. In additional experiments, some cells were treated with 10 uM salmeterol for 24 h following transfection with *IRS-1* shRNA. To determine whether IRS-1 directly regulates apoptotic events in the insulin-signaling pathway in retinal Müller cells, a cell death assay kit was used. In tumor necrosis factor (TNF)α inhibitory studies, cells were treated with 5 ng/ml of TNFα alone for 30 min or 30 min pretreatment with TNFα followed by salmeterol for 4 h.

**Results:**

Müller cells treated with 5 ng/ml TNFα in 25 mM glucose significantly increased phosphorylation of IRS-1^Ser307^. Treatment with the selective beta-2-adrenergic receptor agonist, salmeterol, significantly decreased phosphorylation of IRS-1^Ser307^. Following *IRS-1* shRNA transfection+salmeterol treatment, Bcl-2–associated X protein (Bax) and cytochrome c levels were significantly decreased. Salmeterol+*IRS-1* shRNA also decreased cell death and increased protein levels of B-cell lymphoma-extra large (Bcl-xL), an anti-apoptotic factor.

**Conclusions:**

In these studies, we show for the first time that salmeterol, a beta-2-adrenergic receptor agonist, can reduce retinal Müller cell death through IRS-1 actions. These findings also suggest the importance of IRS-1 in beta-adrenergic receptor signaling in the prevention of cell death in retinal Müller cells.

## Introduction

Over the years, it has been widely accepted that changes that occur in the diabetic retina occur in response to a variety of insults, including high glucose, oxidative stress, and increased expression of inflammatory markers [1-11]. During the initial stages of diabetic retinopathy, Müller cells become activated and express increased glial fibrillary acidic protein levels in diabetes [4,5,11-15]. This increase in glial fibrillary acidic protein levels signals a transition of Müller cells from a quiescent to a reactive state, causing a dysfunction in the regulation of inflammatory markers, glucose transport, oxidative stress, growth factors, and cell survival [4,5,11,15-18]. In diabetic retinopathy, the regulation of insulin signaling, specifically that of insulin receptor substrate (IRS)-1), is not well understood. IRS-1 is a 180 kDa downstream substrate of the insulin receptor and plays a central role in both insulin and insulin-like growth factor (IGF-1) signaling [19-23]. IRS-1 has been shown to have numerous sites for phosphorylation by serine, threonine, and tyrosine, with some sites serving to propagate insulin/IGF-1 receptor signaling, while other residues inhibit insulin/IGF-1 signaling. Tyrosine phosphorylation of IRS-1 is known to be an important step in the propagation of the insulin/IGF-1 signal, while the role of serine and threonine phosphorylation of IRS-1 has recently become of more significance as a component of insulin resistance, since decreased insulin/IGF-1 signaling is likely a key factor in diabetes [19-23]. One of the serine residues on IRS-1 that has been suggested to serve an inhibitory role in insulin signaling is serine 307 [19,23,24]. Previous studies have shown that increases in the phosphorylation of IRS-1^Ser307^ causes decreased insulin receptor signaling, resulting in increased in apoptosis in various tissues throughout the body [23-31].

In vitro and in vivo studies have shown that prolonged exposure to a hyperglycemic environment produces several cellular changes, including increased apoptosis [32,33]. Normal regulation of cell death in the mitochondria is tightly controlled by the B-cell lymphoma 2 (Bcl-2) family, both pro- and antiapoptotic members [10,34-39]. In a disease such as diabetic retinopathy, where the hyperglycemic environment causes cellular stress and damage, Bcl-2–associated X protein (Bax), a member of the Bcl-2 family, can become activated and form pores as a passage for other proapoptotic proteins to be released [10,34-39]. Release of proteins, such as cytochrome c, along with increased Bax levels results in cell death through increased levels of key caspases. In contrast, B-cell lymphoma-extra large (Bcl-xL), an antiapoptotic member of the Bcl-2 family, is known to prevent cell death by inhibiting activation of the proapoptotic proteins [35,37-39]. These changes have been well studied in other diseases, as well as other cell types in diabetic retinopathy [34,36]. However, the regulation of apoptotic proteins in retinal Müller cells is not well characterized. Furthermore, the potential role for IRS-1 in this pathway in the regulation of Bax, cytochrome c, and Bcl-xL has not been investigated.

In this investigation, we hypothesize that silencing the expression of IRS-1 will demonstrate that IRS-1 directly regulates specific apoptotic markers in retinal Müller cells. Additionally, since we have previously demonstrated that beta-adrenergic receptors can decrease tumor necrosis factor (TNF)α levels [40], and TNFα is known to increase IRS-1^Ser307^, we hypothesize that salmeterol, a beta-2-adrenergic receptor agonist, requires IRS-1 actions to decrease apoptosis of retinal Müller cells.

## Methods

### Müller cell culture

Rat retinal Müller cells (rMC-1) were cultured and passaged in Dulbecco's Modified Eagle Medium (DMEM) medium (HyClone, Logan, UT) containing 5 mM glucose (normal glucose) or 25 mM glucose (high glucose), 10% fetal bovine serum (FBS), and 2 mM L-glutamine. Once the cells reached 80% confluency, the concentration of FBS was decreased from 10% to 2% in 25 mM media starved cells. Cells remained in this starved environment for 18–24 h to reduce any serum effects from the medium. Immediately after starvation, cells were treated with 10 μM salmeterol (beta-2-adrenergic receptor agonist) dissolved into high glucose medium for 6 h. Additionally, a specific number of dishes were used as untreated controls for both treatments in both 25 mM glucose and 5 mM glucose for the duration of the treatment. Following treatment, cells were harvested and pelleted in lysis buffer.

### Tumor necrosis factor–α inhibitory studies

In TNFα inhibitory studies, cells were treated with 5 ng/ml of TNFα alone for 30 min or 30 min pretreatment with TNFα followed by 10 μM salmeterol for 4 h. Immediately after treatments, cells were lysed with lysis buffer (1.58 g Tris base, 150 ml sterile water, 1.80 g NaCl, 20 ml 10% Igepal-40, 5 ml 10% Na-deoxycholate, 2 ml 100 mM EDTA, and 1 μg protease inhibitors (all ingredients for lysis buffer; Sigma, Sigma-Aldrich Corp, St. Louis, MO) and harvested at each of the treatment time points.

### shRNA library construction

The sequence for each of the 21 bp shRNA constructs was designed using Invitrogen Block-iT RNA designer™ (Invitrogen, Carlsbad, CA). The sequence for rat *IRS-1* (accession # NM_012969) was 5'-CGA GTT CTG GAT GCA AGT GGA and the sequence of the scrambled shRNA was 5'-GAC GAA CCC CTG TTC CGA ATG. The Mir algorithm was used to design double-stranded cDNAs. For rat *IRS-1*, the sequence of the forward primer was 5′-TGC TGT CCA CTT GCA TCC AGA ACT CG GTT TTG GCC ACT GAC TGA CCG AGT TCT ATG CAA GTG GA-3′ and its complementary strand was 5′-CCT GTC CAC TTG CAT AGA ACT CGG TCA GTC AGT GGC CAA AAC CGA GTT CTG GAT GCA AGT GGA C-3′. These synthetic oligo constructs were hybridized and cloned into BLOCK-iT™ Pol II miR RNAi Expression Vector with EmGFP. Each plasmid was grown on agar plates containing 50 µg/ml of spectinomycin. Colonies were selected and sequenced to verify insert sequence, and then a large plasmid preparation was made using Qiagen kits (Qiagen, Baltimore, MD). Upon transient transfection into cells, expression of the shRNA was monitored by green fluorescent protein (GFP) fluorescence (λex=488 nm, λem=520 nm).

To determine the effect of transient expression of 5 μg of each shRNA/60 mm plate on its target, we probed total RNA by reverse transcriptase (RT)–PCR or protein by western blotting using the anti-IRS-1 antibody (SC-559; Santa Cruz Biotechnology, Santa Cruz, CA). For the RT–PCR procedure, first-strand cDNA synthesis was performed using the Transcriptor First-Strand cDNA Synthesis Kit from (Roche Diagnostics, Indianapolis, IN) using 62 ng of RNA per assay. The RT–PCR primers were designed using a web-based design center (Universal prolibrary). The mRNA level for each protein was quantified using the Universal prolibrary of short hydrolysis-locked nucleic acid probes in combination with the primers. The quantification of mRNA was accomplished using the Roche Lightcycler 480 Real-time PCR system and software (Roche diagnostics).

### RNA interference transfection

For shRNA studies, cells were passaged and cultured until 80% confluency, at which time cells were transfected with shRNA to silence IRS-1 using lipofectamine for 24 h. For *IRS-1* shRNA + salmeterol studies, following the 24 h of transfection, cells were treated with 10 μM salmeterol for an additional 6 h. Cells that were designated as *IRS-1* shRNA alone were harvested with no further treatment following the 24 h transfection period. For scrambled shRNA studies, cells were transfected with scrambled shRNA using lipofectamine for 24 h.

### Western blot analysis

Cells stored in lysis buffer containing protease inhibitors (leupeptin 1 μg/ml, aprotinin 1 μg/ml) were homogenized, sonicated, and protein concentrations were determined by Bradford assay (Thermo Fisher Scientific, Rockford, IL). Denaturing sample buffer (2× glass distilled water [GDW], 1M Tris-HCL pH 6.8, 30% glycerol, β-mercaptoethanol, 0.05% bromophenol blue, and 0.125 g recrystallized sodium dodecyl sulfate [SDS]) was added to 30–50 μg of protein and loaded onto 10%–20% precast tris-glycine gels (Invitrogen, Carlsbad, CA) for separation, followed by transfer to nitrocellulose membranes. Membranes were blocked overnight at 4 °C with 5% BSA and with the following primary antibodies: IRS-1 Ser307 (diluted 1:500; Cell Signaling, Beverly, MA), total IRS-1 (diluted 1:500; Cell Signaling), Bcl-xL (diluted 1:500; Cell Signaling), Bax (diluted 1:500; Cell Signaling), Akt (diluted 1:500; Cell Signaling), and cytochrome c (diluted 1:500; Cell Signaling). All blots were washed and then incubated at room temperature with the appropriate secondary antibodies conjugated to horseradish peroxidase at 1:5,000 dilutions. Following secondary antibodies, blots were washed and placed into enhanced chemiluminescence (ECL) reagent (Pierce, Rockford, IL) for chemiluminescent detection using the Kodak ImageStation 4000MM (Rochester, NY). Mean densitometry of immunoreactive bands was assessed using Kodak software, and results were expressed in densitometric units and compared to control groups for each individual experiment.

### Cell death assays

Cell death was assessed in rMC-1 cells using a cell death assay kit (Roche Diagnostics) following the manufacturer’s instructions. Müller cell lysates were transferred into a streptavidin-coated microplate that was provided by manufacturer. Mixtures of anti-histone-biotin and anti-DNA-POD antibodies were added to the cells for a short incubation periods. During these periods, the mixture was allowed to bind the nucleosomes and histones of cells plated. Following incubation, washing took place to remove any antibodies that did not bind during the incubation period. Following these steps, plates were measured according to manufacturer's instructions. This assay measures histone-associated DNA fragments in a quantitative manner in retinal Müller cells. Analysis from these experiments was performed using absorbance values obtained at the appropriate wavelength, followed by statistics using Prism 4.0 with comparisons between the control and treatment groups using Mann–Whitney as a post-hoc test with p<0.05 being accepted as significant.

### Statistical analysis

All statistical analyses for these investigations were obtained using Prism 4.0b software. Nonparametric tests were conducted for cell culture experiments due to the small sample size for each experiment. For all experiments, the 5 mM (#) and 25 mM (*) glucose samples (controls) were compared to *IRS-1* shRNA treatment groups and salmeterol treatment groups using a Mann–Whitney U test, with p<0.05 considered as significantly different. Additionally, a separate comparison was conducted with *IRS-1* shRNA treatment + salmeterol versus salmeterol treatment alone ($).

## Results

### Salmeterol prevents phosphorylation levels of IRS-1^Ser307^ induced by tumor necrosis factor–α

**Figure 1:**
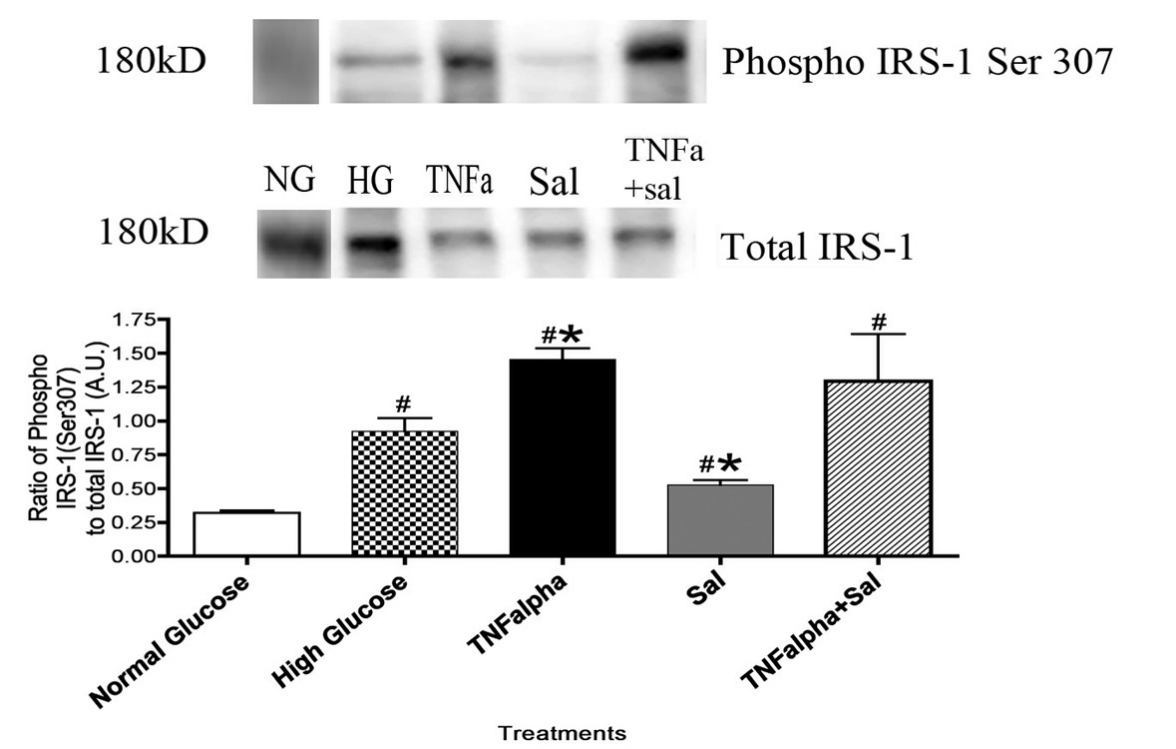
Ratio of insulin receptor substrate (IRS)-1^Ser 307^ in Müller cells. A phosphorylation of IRS-1^Ser307^ is significantly increased in Müller cells following treatment with tumor necrosis factor–α, but treatment with beta-2-adrenergic receptor agonist, salmeterol significantly decreases phosphorylation levels. Significance was determined by one-tailed, nonparametric Mann–Whitney test on western blot data (*p<0.05 versus high glucose, n=4, #p<0.05 versus normal glucose, n=4).

It is known that TNFα preferentially phosphorylates IRS-1^Ser307^ in other cell types [23,26,27,30,31]; we wanted to see if the same mechanism occurs in retinal Müller cells. Following treatment with salmeterol, western blot analysis revealed that phosphorylation of IRS-1^Ser307^ was significantly decreased as compared to cells without treatment or with TNFα-only treatment (Figure 1, *p<0.05 versus not treated, # p<0.05 versus TNFα alone).

### Silencing of IRS-1 decreases total Akt levels

**Figure 2:**
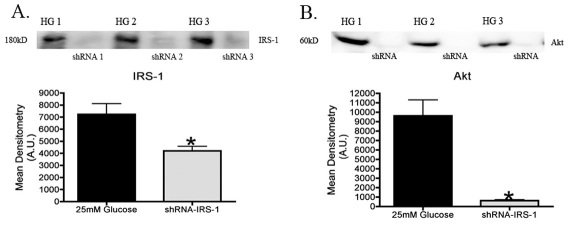
Verification of insulin receptor substrate (IRS)-1 shRNA knockdown. **A**: Mean densitometry and representative blot of IRS-1 levels following transfection of shRNA (*IRS-1*) in rat Müller cells. Mean densitometry was done for each blot which consisted of taking the mean optical densities of 4 different western blots for each protein analyzed. **B**: Mean Densitometry of Akt levels following transfection of shRNA (*IRS-1*) in Müller cells. Significance was determined by Mann–Whitney test (*p<0.05 versus 25 mM, n=5).

Previous data has shown that increases in tyrosine phosphorylation of insulin receptor results in increased Akt phosphorylation via IRS-1. Knockdown of *IRS-1* with shRNA (Figure 2A, *p<0.05 versus 25 mM glucose) showed a significant decrease in total Akt levels (Figure 2B, *p<0.05 versus 25 mM glucose) cultured in a hyperglycemic environment. These results suggest that IRS-1 signals to Akt in retinal Müller cells.

### Loss of IRS-1 increases cell death in retinal Müller cells

**Figure 3:**
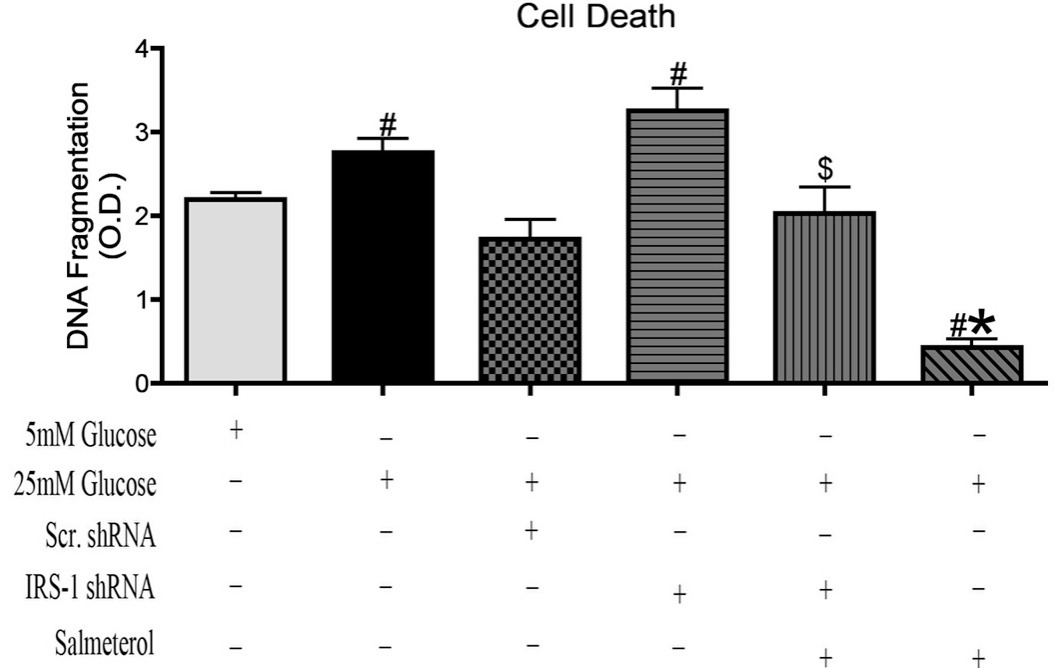
Cell death in insulin receptor substrate (IRS)-1 knockdown. **A**: Cell Death ELISA of rat Müller cells transfected with *IRS-1* shRNA alone and *IRS-1* shRNA + beta-2-adrenergic receptor agonist, salmeterol for 6 h. Transfection with *IRS-1* shRNA significantly increased cell death levels versus 5 mM glucose. Treatment with salmeterol (SALM) alone in Müller cells significantly decreased levels of cell versus 5 mM glucose and 25 mM glucose. Statistical significance was determined Mann–Whitney test (*p<0.05 versus 25 mM glucose, #p<0.05 versus 5 mM glucose, $p<0.05 versus SALM n=5 for ELISA assay).

Treatment of cells with salmeterol alone prevented cell death in retinal Müller cells (Figure 3, p<0.05 versus 25 mM glucose). Cell death analyses showed a significant increase in response to silencing of IRS-1 in cells cultured in high glucose versus normal glucose (Figure 3, #p<0.05 versus 5 mM glucose). Salmeterol + IRS-1 shRNA showed a significant increase in cell death compared to salmeterol alone (Figure 3, $p<0.05 versus salmeterol alone), suggesting that beta-adrenergic receptors signal through IRS-1 to reduce cell death in retinal Müller cells.

### Silencing IRS-1 increases cytochrome C levels in retinal Müller cells

**Figure 4:**
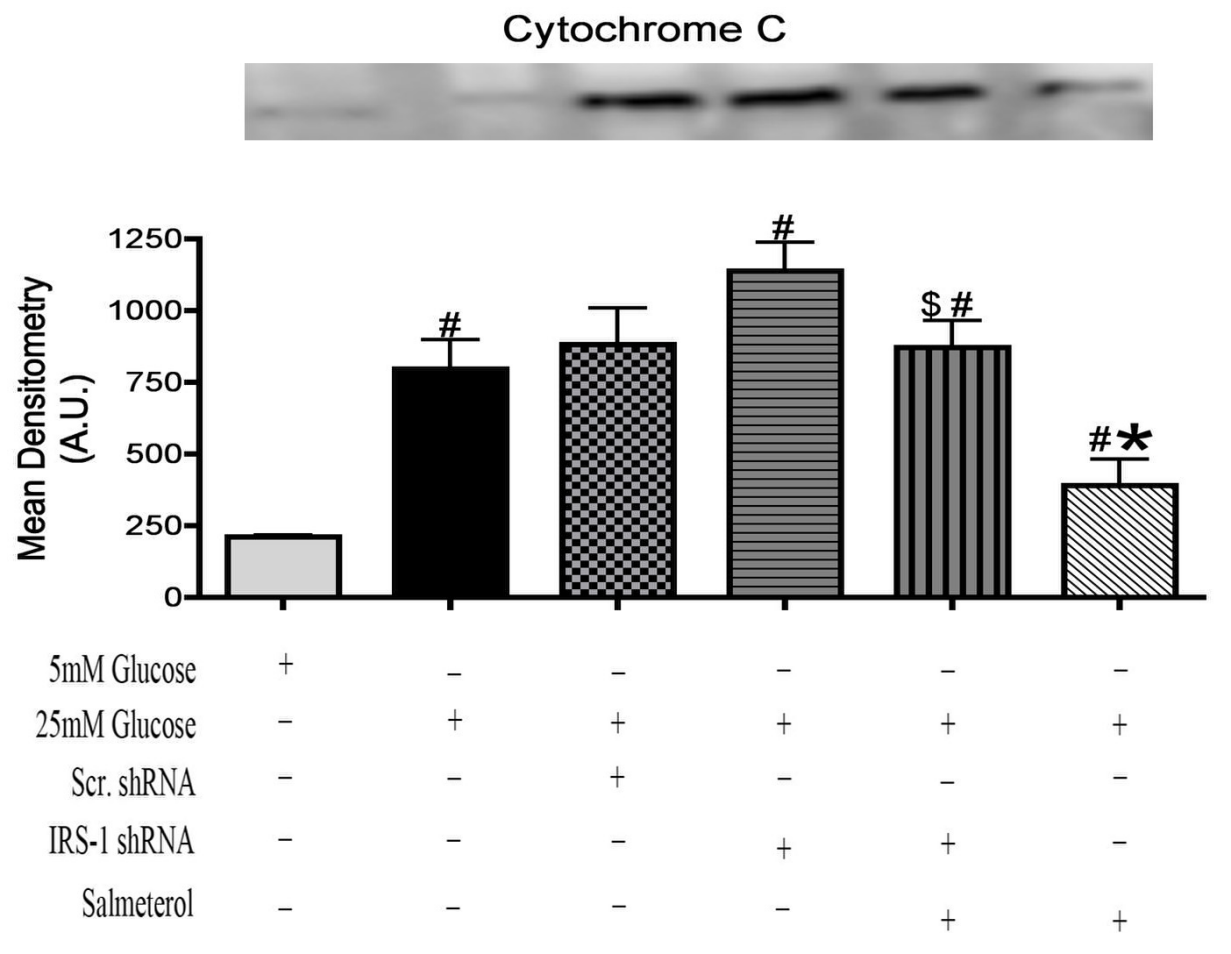
Levels of cytochrome C increased in insulin receptor substrate (IRS)-1 knockdown. Equal loading was verified using ponceau staining. Mean densitometry was done for each blot which consisted of taking the mean optical densities of 4 different western blots for each protein analyzed. Mean densitometry displayed a significant increase in cytochrome c levels cultured in 25 mM glucose versus 5 mM glucose samples. Knockdown of IRS-1 protein significantly increased cytochrome C levels versus 5 mM and 25 mM glucose samples. Western blot data showing that treatment with salmeterol significantly decreased levels of proapoptotic cytochrome C (*p<0.05 versus 5 mM glucose [NT], $p<0.05 versus salmeterol n=4 for western blot).

Previous studies have suggested the mitochondria as a key regulator of apoptosis, with excess production of superoxides within the mitochondria initiating cytochrome c being released from the cytosol to begin the cascade of apoptotic signaling [35,37-39]. Our current investigation shows that prolonged exposure of retinal Müller cells to hyperglycemia results in the excess release of cytochrome C when compared to retinal Müller cells cultured in normal glycemic conditions (Figure 4, *p<0.05 versus 5 mM glucose). Western blot analysis further shows that salmeterol alone treatment significantly reduced cytochrome C levels, with the effect lessened when salmeterol was combined with *IRS-1* shRNA (Figure 4, $p<0.05 versus salmeterol alone). Taken together, these results suggest that active IRS-1 is required for salmeterol to reduce cytochrome C levels in retinal Müller cells cultured in a hyperglycemic environment.

### Absence of IRS-1 causes an increase in Bax levels

**Figure 5:**
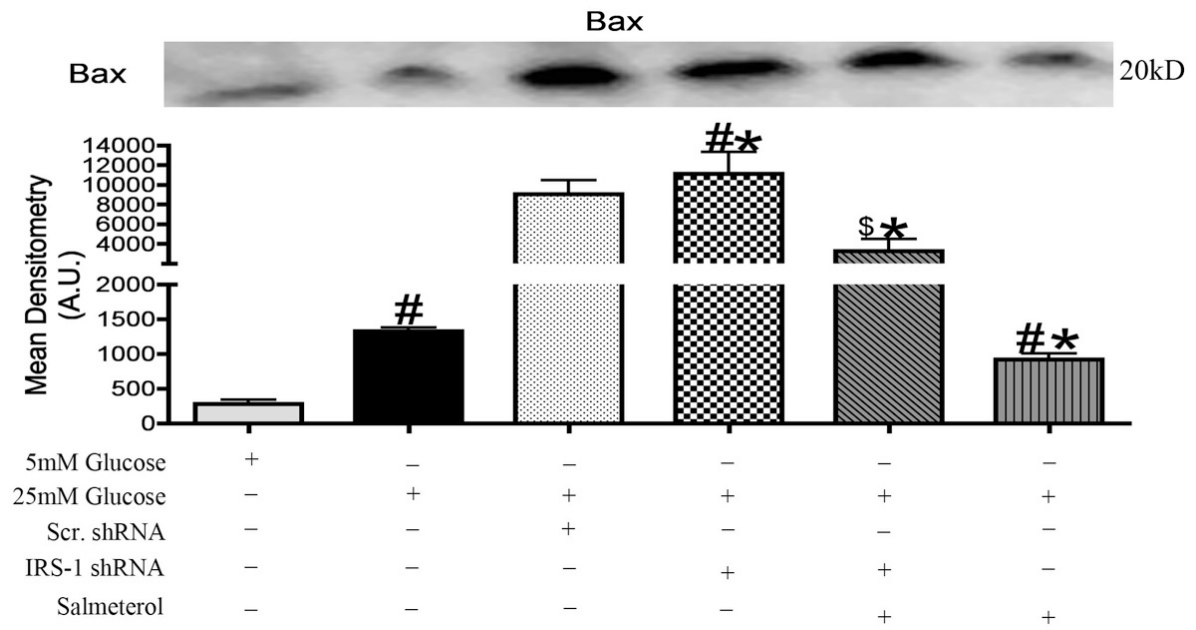
Bax protein levels increased in insulin receptor substrate (IRS)-1 knockdown. Protein levels of Bax were significantly increased 25 mM glucose versus 5 mM glucose samples. Transfection of *IRS-1* shRNA in Müller cells significantly increased levels of pro-apoptotic Bax. Salmeterol (10 uM) significantly decreased Bax levels activity after 6 h of treatment. Significance was determined by the Mann–Whitney test (*p<0.05 versus 25 mM glucose, $p<0.05 versus salmeterol n=4, #p<0.05 versus 5 mM glucose, n=4 for western blot). Equal loading was verified using ponceau staining. Mean densitometry was done for each blot which consisted of taking the mean optical densities of 4 different western blots for each protein analyzed.

In addition to cytochrome C, we also investigated another member of the Bcl-2 family, Bax. Western blot analyses showed significant increases in Bax protein levels in high glucose samples compared to normal glucose samples (Figure 5, #p<0.05 versus 5 mM glucose). Stimulation with salmeterol showed that salmeterol could only reduce Bax when IRS-1 was active (Figure 5, $p<0.05 versus salmeterol alone). These findings were in agreement with previous findings that suggested increased Bax levels in a hyperglycemic environment [2,36]; however, to our knowledge, these results are the first to link beta-adrenergic receptors and IRS-1 to Bax levels in retinal Müller cells.

### Antiapoptotic Bcl-xL is reduced with the loss of IRS-1

**Figure 6:**
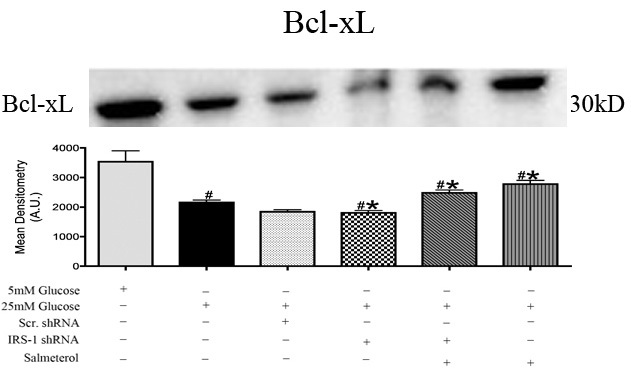
Anti-apoptotic Bcl-xL levels decreased in insulin receptor substrate (IRS)-1 knockdown. Western blot analysis showing significantly decreased levels of Bcl-xL versus 5 mM glucose and 25 mM glucose. Salmeterol treatment significantly increased levels of Bcl-xL toward basal levels. Equal loading was verified using ponceau staining. Mean densitometry was done for each blot which consisted of taking the mean optical densities of 4 different western blots for each protein analyzed. Significance was determined by one-tailed, nonparametric Mann–Whitney tests on western blot data (*p<0.05 versus 25 mM Glucose, $p<0.05 versus salmeterol n=4, #p<0.05 versus 5 mM Glucose, n=4).

Previous investigations suggest that IRS-1 plays a role in antiapoptotic activities through proper control of the members of the Bcl-2 family, such as Bcl-xL [2,36,41,42]. Mechanisms of the interplay between IRS-1 and Bcl-xL are unknown [42]. Protein levels of Bcl-xL were significantly decreased in high glucose samples compared to normal glucose samples (Figure 6, #p<0.05 versus 5 mM glucose), suggesting that high glucose promotes an apoptotic environment. Treatment with salmeterol alone significantly increased Bcl-xL protein levels compared to high glucose samples (Figure 6, *p<0.05 versus 25 mM glucose). Transfection of retinal Müller cells with *IRS-1* shRNA showed a significant decrease in protein levels of antiapoptotic Bcl-xL in a hyperglycemic environment (Figure 6, #p<0.05 versus 5 mM glucose). These findings suggest beta-adrenergic receptor modulation of antiapoptotic Bcl-xL, which would promote an antiapoptotic environment.

## Discussion

Previously, our laboratory has suggested that hyperglycemia can increase inflammatory cytokine production in retinal Müller cells [40]. The increase in inflammatory cytokines, such as TNFα, in a hyperglycemic environment was significantly reduced when cells were treated with the nonselective beta-adrenergic receptor agonist, isoproterenol [40]. These studies further suggested the presence of beta-adrenergic receptors on retinal Müller cells, and that the loss of beta adrenergic receptor signaling may be involved in the increased inflammatory markers observed in hyperglycemia [40]. Recent studies by other groups have identified a potential role for inflammation in the regulation of key signaling pathways in diabetes [4,23-31,42-46]. These studies have suggested that TNFα may mediate changes in insulin receptor signaling by targeting downstream signaling proteins, such as the IRS complex, to produce pathologic changes [4,23-31,42-46].

The IRS complex proteins are responsible for mediating the downstream actions of the insulin receptor. The IRS complex consists of IRS 1–4, with each substrate playing a significant role in the body; however, animal studies have shown that a vast majority of insulin actions signal through IRS-1 and IRS-2 [47]. The amino acid sequence of IRS-1 possesses a unique signaling mechanism of tyrosine phosphorylation sites and serine phosphorylation sites [23,28-31,42,44,46,48] to regulate cellular actions. Phosphorylation of various tyrosine sites (Y^99^, Y^1150^, Y^1151^) and several serine sites (Ser^265^, Ser^302^, Ser^325^, Ser^358^) increase the downstream signaling mediated by IRS-1. In contrast, other serine residues (such as Ser^307^, Ser^636^, Ser^639^) have been shown to inhibit signaling downstream of IRS-1, suggesting that IRS-1 phosphorylation may be a key regulator for activation or inhibition of a multitude of signaling cascades.

Based on the literature on other cell types, with the onset of diabetes, TNFα preferentially phosphorylates Ser^307^ on IRS-1 [6,48,49]. Phosphorylation of IRS-1^Ser307^ can play an inhibitory role in insulin/IGF-1 receptor signal transduction, potentially leading to the increased apoptosis noted in the diabetic retina [6,48,49]. The present findings in Müller cells (Figure 1) confirm work in adipose tissue cells, suggesting that TNFα negatively regulates insulin receptor signaling by phosphorylating Ser^307^ on IRS-1 to inhibit insulin action [6,48]. In these studies, we began using a selective beta-2-adrenergic receptor agonist, salmeterol, to selectively stimulate the beta-2-adrenergic receptor, since we have recently found that this receptor is active in retinal Müller cells. Our findings in this study with salmeterol demonstrate that beta-2-adrenergic receptor stimulation may inhibit cytokine release in retinal Müller cells cultured in a hyperglycemic environment, resulting in reduced IRS-1^Ser307^ phosphorylation, thereby leading to decreased apoptosis.

Additionally, we investigated whether beta-adrenergic receptors regulate apoptosis of retinal Müller cells through IRS-1 signaling. Since we know that beta-adrenergic receptors can decrease cell death in a high glucose environment [50], we sought to determine whether modulation of IRS-1 was involved. Knockdown of IRS-1 showed a significant increase in cell death compared to samples in 5 mM glucose, but stimulation of the beta-2-adrenergic receptor with salmeterol prevented cell death through IRS-1 in a hyperglycemic environment on retinal Müller cells.

Several factors can influence the increase in apoptosis. An imbalance in the expression of antiapoptotic versus proapoptotic members of the Bcl-2 family within the mitochondria of retinal Müller cells is one possibility. Retinal Müller cell samples cultured in a 25 mM glucose environment showed a significant increase in cytochrome C and Bax levels compared to samples cultured in 5 mM glucose. We found that activation of cytochrome C and Bax in a hyperglycemic environment was reduced following treatment with salmeterol. Increases in cytochrome C and Bax were also demonstrated with *IRS-1* shRNA + salmeterol, indicative of increased cell death following knockdown of IRS-1 versus cells treated with salmeterol alone. Our results suggest that beta-adrenergic receptors play a specific role in the regulation of key apoptotic markers through alterations in IRS-1 levels. In support of this finding, we also found that high glucose decreased antiapoptotic Bcl-xL, but treatment with salmeterol significantly increased Bcl-xL in a hyperglycemic environment. Decreased Bcl-xL levels were also observed in *IRS-1* shRNA +salmeterol treatments, suggesting that the antiapoptotic effects of Bcl-xL restored with treatment of salmeterol required IRS-1 for activation.

To our knowledge, our research is the first to report that salmeterol, a beta-2-adrenergic receptor agonist, can reduce cell death activity in retinal Müller cells using IRS-1 signaling. However, our results are not in agreement with previous results that suggest that IRS-2 is the key mediator of cell death in whole retinal samples [44,45]. The discrepancies in our findings in relation to those of previous studies likely stem from the fact that we concentrated solely on in vitro studies using retinal Müller cells. Previous studies have dealt with in vivo and ex vivo experiments using whole retinal samples, which contain a variety of retinal cell types. In other work from our laboratory [50], we have found differences in insulin receptor substrate signaling in retinal endothelial cells, which tend to signal through an IGF-1 receptor/IRS-2-dependent mechanism [50]. Thus, it appears that different cell types in the retina may use different IRS complexes for cellular signaling, which expands the signaling possibilities of retinal cells.

While we recognize that IRS-1 is a key component of insulin signaling, we chose to focus our investigations on beta-adrenergic receptor regulation of apoptosis of retinal Müller cells through the actions of IRS-1 rather than insulin receptor or IGF-1 receptor actions. Future studies may be directed at beta-adrenergic receptor actions and cross talk with insulin signaling.

In summary, these studies demonstrate that retinal Müller cells cultured in an hyperglycemic environment activate several mechanisms leading to increased cell death; 1) the initial mechanism involves increases in phosphorylation of IRS-1^Ser307^, mediated by increased TNFα levels in the diabetic retina [7]; 2) the second mechanism involves significant increases in apoptotic markers Bax and cytochrome C, coupled with a significant decrease in antiapoptotic Bcl-xL. Both mechanisms of cell death were significantly inhibited following treatment with a beta-2-adrenergic receptor agonist, salmeterol. Taken together, these results suggest that beta-adrenergic receptors require active IRS-1 to prevent cell death in retinal Müller cells.

## References

[1] Klein R (1996). Diabetic retinopathy.. Annu Rev Public Health.

[2] Abu-El-Asrar AM, Dralands L, Missotten L, Al-Jadaan IA, Geboes K (2004). Expression of apoptosis markers in the retinas of human subjects with diabetes.. Invest Ophthalmol Vis Sci.

[3] Frank RN (2004). Diabetic retinopathy.. N Engl J Med.

[4] Kondo T, Kahn CR (2004). Altered insulin signaling in retinal tissue in diabetic states.. J Biol Chem.

[5] Bringmann A, Pannicke T, Grosche J, Francke M, Wiedemann P, Skatchkov SN, Osborne NN, Reichenbach A (2006). Muller cells in the healthy and diseased retina.. Prog Retin Eye Res.

[6] Hotamisligil GS (2006). Inflammation and metabolic disorders.. Nature.

[7] Joussen AM, Doehmen S, Le ML, Koizumi K, Radetzky S, Krohne TU, Poulaki V, Semkova I, Kociok N (2009). TNF-alpha mediated apoptosis plays an important role in the development of early diabetic retinopathy and long-term histopathological alterations.. Mol Vis.

[8] Mysona B, Dun Y, Duplantier J, Ganapathy V, Smith S (2009). Effects of hyperglycemia and oxidative stress on the glutamate transporters GLAST and system xc- in mouse retinal Müller glial cells.. Cell Tissue Res.

[9] Tret'iak EB, Syroedova ON, Neuhaus O, Andreeva AV, Antsiferov MB, Mkrtumian AM, Suchkov SV (2010). Cytokines and their role in pathogenesis of diabetic retinopathy.. Vestn Oftalmol.

[10] Trudeau K, Molina AJ, Guo W, Roy S (2010). High glucose disrupts mitochondrial morphology in retinal endothelial cells: implications for diabetic retinopathy.. Am J Pathol.

[11] Curtis TM, Hamilton R, Yong PH, McVicar CM, Berner A, Pringle R, Uchida K, Nagai R, Brockbank S, Stitt AW (2011). Müller glial dysfunction during diabetic retinopathy in rats is linked to accumulation of advanced glycation end-products and advanced lipoxidation end-products.. Diabetologia.

[12] Mizutani M, Gerhardinger C, Lorenzi M (1998). Muller cell changes in human diabetic retinopathy.. Diabetes.

[13] King JL, Guidry C (2004). Muller cell production of insulin-like growth factor-binding proteins in vitro: modulation with phenotype and growth factor stimulation.. Invest Ophthalmol Vis Sci.

[14] Kusner LL, Sarthy VP, Mohr S (2004). Nuclear translocation of glyceraldehyde-3-phosphate dehydrogenase: a role in high glucose-induced apoptosis in retinal Muller cells.. Invest Ophthalmol Vis Sci.

[15] Guidry C, King JL, Mason JO (2009). Fibrocontractive Müller cell phenotypes in proliferative diabetic retinopathy.. Invest Ophthalmol Vis Sci.

[16] Fletcher EL, Phipps JA, Ward MM, Puthussery T, Wilkinson-Berka JL (2007). Neuronal and glial cell abnormality as predictors of progression of diabetic retinopathy.. Curr Pharm Des.

[17] Li Q, Puro DG (2002). Diabetes-induced dysfunction of the glutamate transporter in retinal Muller cells.. Invest Ophthalmol Vis Sci.

[18] Kowluru RA, Tang J, Kern TS (2001). Abnormalities of retinal metabolism in diabetes and experimental galactosemia. VII. Effect of long-term administration of antioxidants on the development of retinopathy.. Diabetes.

[19] Aguirre V, Werner ED, Giraud J, Lee YH, Shoelson SE, White MF (2002). Phosphorylation of Ser307 in insulin receptor substrate-1 blocks interactions with the insulin receptor and inhibits insulin action.. J Biol Chem.

[20] Greene MW, Garofalo RS (2002). Positive and Negative Regulatory Role of Insulin Receptor Substrate 1 and 2 (IRS-1 and IRS-2) Serine/Threonine Phosphorylation.. Biochemistry.

[21] Hartman ME, Villela-Bach M, Chen J, Freund GG (2001). FRAP-Dependent Serine Phosphorylation of IRS-1 Inhibits IRS-1 Tyrosine Phosphorylation.. Biochem Biophys Res Commun.

[22] Luo M, Langlais P, Yi Z, Lefort N, De Filippis EA, Hwang H, Christ-Roberts CY, Mandarino LJ (2007). Phosphorylation of human insulin receptor substrate-1 at Serine 629 plays a positive role in insulin signaling.. Endocrinology.

[23] Luo M, Reyna S, Wang L, Yi Z, Carroll C, Dong LQ, Langlais P, Weintraub ST, Mandarino LJ (2005). Identification of insulin receptor substrate 1 serine/threonine phosphorylation sites using mass spectrometry analysis: regulatory role of serine 1223.. Endocrinology.

[24] Aguirre V, Uchida T, Yenush L, Davis R, White MF (2000). The c-Jun NH(2)-terminal kinase promotes insulin resistance during association with insulin receptor substrate-1 and phosphorylation of Ser(307).. J Biol Chem.

[25] Boura-Halfon S, Zick Y (2009). Phosphorylation of IRS proteins, insulin action, and insulin resistance.. Am J Physiol Endocrinol Metab.

[26] Hotamisligil GS, Budavari A, Murray D, Spiegelman BM (1994). Reduced tyrosine kinase activity of the insulin receptor in obesity-diabetes. Central role of tumor necrosis factor-alpha.. J Clin Invest.

[27] Hotamisligil GS, Peraldi P, Budavari A, Ellis R, White MF, Spiegelman BM (1996). IRS-1-mediated inhibition of insulin receptor tyrosine kinase activity in TNF-alpha- and obesity-induced insulin resistance.. Science.

[28] Paz K, Hemi R, LeRoith D, Karasik A, Elhanany E, Kanety H, Zick Y (1997). A molecular basis for insulin resistance. Elevated serine/threonine phosphorylation of IRS-1 and IRS-2 inhibits their binding to the juxtamembrane region of the insulin receptor and impairs their ability to undergo insulin-induced tyrosine phosphorylation.. J Biol Chem.

[29] Paz K, Liu YF, Shorer H, Hemi R, LeRoith D, Quan M, Kanety H, Seger R, Zick Y (1999). Phosphorylation of insulin receptor substrate-1 (IRS-1) by protein kinase B positively regulates IRS-1 function.. J Biol Chem.

[30] Sykiotis GP, Papavassiliou AG (2001). Serine phosphorylation of insulin receptor substrate-1: a novel target for the reversal of insulin resistance.. Mol Endocrinol.

[31] White MF (2002). IRS proteins and the common path to diabetes.. Am J Physiol Endocrinol Metab.

[32] Kowluru RA (2005). Diabetic Retinopathy: Mitochondrial Dysfunction and Retinal Capillary Cell Death.. Antioxid Redox Signal.

[33] Kowluru RA, Kennedy A (2001). Therapeutic potential of anti-oxidants and diabetic retinopathy.. Expert Opin Investig Drugs.

[34] Hasnan J, Yusof MI, Damitri TD, Faridah AR, Adenan AS, Norbaini TH (2010). Relationship between apoptotic markers (Bax and Bcl-2) and biochemical markers in type 2 diabetes mellitus.. Singapore Med J.

[35] Hetz C (2010). BCL-2 protein family. Essential regulators of cell death. Preface.. Adv Exp Med Biol.

[36] Khalfaoui T, Basora N, Ouertani-Meddeb A (2010). Apoptotic factors (Bcl-2 and Bax) and diabetic retinopathy in type 2 diabetes.. J Mol Histol.

[37] Soriano ME, Scorrano L (2010). The interplay between BCL-2 family proteins and mitochondrial morphology in the regulation of apoptosis.. Adv Exp Med Biol.

[38] Lindsay J, Esposti MD, Gilmore AP (2011). Bcl-2 proteins and mitochondria–specificity in membrane targeting for death.. Biochim Biophys Acta.

[39] Zhou F, Yang Y, Xing D (2011). Bcl-2 and Bcl-xL play important roles in the crosstalk between autophagy and apoptosis.. FEBS J.

[40] Walker RJ, Steinle J (2007). Role of Beta-adrenergic Receptors in Inflammatory Marker Expression in Muller Cells.. Invest Ophthalmol Vis Sci.

[41] Kern TS, Du Y, Miller CM, Hatala DA, Levin LA (2010). Overexpression of Bcl-2 in vascular endothelium inhibits the microvascular lesions of diabetic retinopathy.. Am J Pathol.

[42] Ueno H, Kondo E, Yamamoto-Honda R, Tobe K, Nakamoto T, Sasaki K, Mitani K, Furusaka A, Tanaka T, Tsujimoto Y, Kadowaki T, Hirai H (2000). Association of insulin receptor substrate proteins with Bcl-2 and their effects on its phosphorylation and antiapoptotic function.. Mol Biol Cell.

[43] Baltensperger K, Karoor V, Paul H, Ruoho A, Czech MP, Malbon CC (1996). The beta-adrenergic receptor is a substrate for the insulin receptor tyrosine kinase.. J Biol Chem.

[44] Reiter CE, Sandirasegarane L, Wolpert EB, Klinger M, Simpson IA, Barber AJ, Antonetti DA, Kester M, Gardner TW (2003). Characterization of insulin signaling in rat retina in vivo and ex vivo.. Am J Physiol Endocrinol Metab.

[45] Reiter CE, Wu X, Sandirasegarane L, Nakamura M, Gilbert KA, Singh RS, Fort PE, Antonetti DA, Gardner TW (2006). Diabetes Reduces Basal Retinal Insulin Receptor Signaling.. Diabetes.

[46] Valverde AM, Mur C, Pons S, Alvarez AM, White MF, Kahn CR, Benito M (2001). Association of insulin receptor substrate 1 (IRS-1) y895 with Grb-2 mediates the insulin signaling involved in IRS-1-deficient brown adipocyte mitogenesis.. Mol Cell Biol.

[47] Withers DJ, White M (2000). Perspective: The insulin signaling system–a common link in the pathogenesis of type 2 diabetes.. Endocrinology.

[48] Taniguchi CM, Emanuelli B, Kahn CR (2006). Critical nodes in signalling pathways: insights into insulin action.. Nat Rev Mol Cell Biol.

[49] Lorenzo M, Fernandez-Veledo S, Vila-Bedmar R, Garcia-Guerra L, De Alvaro C, Nieto-Vazquez I (2008). Insulin resistance induced by tumor necrosis factor-alpha in myocytes and brown adipocytes.. J Anim Sci.

[50] Panjala SR, Steinle J (2011). Insulin and β-adrenergic Receptors Inhibit Retinal Endothelial Cell Apoptosis Through Independent Pathways.. Neurochem Res.

